# Anatomical and anastomotic viability indexes for stratifying the risk of anastomotic leakage in esophagectomy with retrosternal reconstruction

**DOI:** 10.1002/ags3.12693

**Published:** 2023-05-16

**Authors:** Keita Takahashi, Katsunori Nishikawa, Yuichiro Tanishima, Yoshitaka Ishikawa, Takanori Kurogochi, Masami Yuda, Akira Matsumoto, Fumiaki Yano, Toru Ikegami, Ken Eto

**Affiliations:** ^1^ Department of Gastrointestinal Surgery Jikei University School of Medicine Tokyo Japan

**Keywords:** anastomotic leakage, anastomotic viability index, anatomical index, esophagectomy

## Abstract

**Background:**

Risk prediction of anastomotic leakage using anatomical and vascular factors has not been well established. This study aimed to assess the anatomical and vascular factors affecting the hemodynamics of the gastric conduit and develop a novel risk stratification system in patients undergoing esophagectomy with retrosternal reconstruction.

**Methods:**

This retrospective cohort study analyzed 202 patients with esophageal cancer who underwent subtotal esophagectomy with gastric tube retrosternal reconstruction between January 2008 and December 2020. Risk factors for anastomotic leakage (AL), including the anatomical index (AI) and anastomotic viability index (AVI), were evaluated using a logistic regression model.

**Results:**

According to the logistic regression model, the independent risk factors for AL were preoperative body mass index ≥23.6 kg/m^2^ (odds ratio [OR], 7.97; 95% confidence interval [CI], 2.44–26.00; *P <* 0.01), AI <1.4 (OR, 23.90; 95% CI, 5.02–114.00; *P <* 0.01), and AVI <0.62 (OR, 8.02; 95% CI, 2.57–25.00; *P <* 0.01). The patients were stratified into four AL risk groups using AI and AVI as follows: low‐risk group (AI ≥1.4, AVI ≥0.62 [2/99, 2.0%]), intermediate low‐risk group (AI ≥1.4, AVI <0.62 [2/29, 6.9%]), intermediate high‐risk group (AI <1.4, AVI ≥0.62 [8/53, 15.1%]), and high‐risk group (AI <1.4, AVI <0.62 [11/21, 52.4%]).

**Conclusion:**

The combination of AI and AVI strongly predicted AL. Additionally, the use of AI and AVI enabled the stratification of the risk of AL in patients who underwent esophagectomy with retrosternal reconstruction.

## Graphical abstract

This study revealed that anastomotic leakage after esophagectomy with retrosternal reconstruction was strongly affected by anatomical and anastomotic viability indices. Patients could be stratified into low‐, intermediate‐, and high‐risk groups. High‐risk patients may be a suitable alternative.

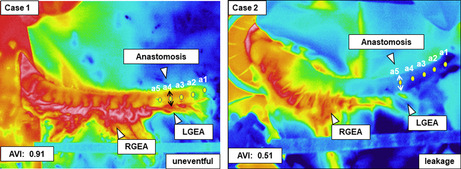

## 1 INTRODUCTION

Esophageal cancer is the seventh most prevalent malignancy and sixth leading cause of cancer‐related deaths worldwide.^1^ Despite recent progress in multidisciplinary treatment, esophagectomy remains an essential curative therapy.^2^, ^3^ Esophagectomy is an invasive and complicated surgical procedure that generally results in higher postoperative morbidities than those of other surgical treatments for malignant disease.^4^, ^5^

Anastomotic leakage (AL) is a critical complication after esophageal cancer surgery and has a negative impact on a patient's prognosis.^6^, ^7^ A nationwide study revealed that the incidence of AL was 13.3% in patients undergoing surgery for esophageal cancer,^4^ which was notably higher than that in other gastrointestinal surgeries. Several previous studies have reported that poor vascularity of the gastric conduit was significantly associated with AL.^8^ Specifically, we previously showed that the anastomotic viability index (AVI) derived from thermal imaging (TI) was a valuable marker for AL because TI could be used to quantitatively evaluate the blood flow of the gastric conduit during surgery.^9^

Generally, the three esophageal reconstruction routes after esophagectomy for esophageal cancer surgeries, including the posterior mediastinal (PM), retrosternal, and subcutaneous routes, have been used at the surgeons' discretion. Several studies have investigated suitable reconstruction routes, but no consensus has been reached. Unlike in Western countries, squamous cell carcinoma (SCC) is Japan's most dominant esophageal cancer subtype and is known to metastasize in the mediastinal lymph nodes or cause relapse in patients. Therefore, the retrosternal route is preferred for esophageal replacement to prevent malignant infiltration into organs reconstructed because of relapsed tumors or damage from postoperative radiation therapy. In a nationwide Japanese study, nearly 65% of patients with esophageal cancer subsequently underwent retrosternal reconstruction.^10^ Previous reports have revealed that the space of the thoracic inlet was strongly associated with AL progression in patients who had undergone retrosternal reconstruction.^11^, ^12^, ^13^, ^14^ On the basis of these findings, we hypothesized that the anatomical factors affecting external compression at the thoracic inlet against the gastric conduit and AVI corresponding to gastric conduit vascularity should be powerful predictors of AL for retrosternal reconstruction.

This study aimed to assess the effect of anatomical factors and AVI in patients planning to undergo esophagectomy with retrosternal reconstruction, which could potentially reduce AL. We also examined the value of patient stratification to predict their risk of AL and facilitate selection of either the retrosternal or PM route for esophagectomy.

## 2 PATIENTS AND METHODS

### 2.1 Patients

We included 246 patients with esophageal cancer who underwent subtotal esophagectomy with gastric tube reconstruction at the Jikei University School of Medicine between January 2008 and December 2020. After excluding patients who underwent PM reconstruction (*n* = 25), subcutaneous reconstruction (*n* = 15), and those without available computed tomography (CT) data (*n* = 2), the remaining 202 patients were included in the study (Figure S1). Data on the patient background characteristics, histopathological findings, intraoperative findings, tumor stage, and postoperative complications were obtained from their medical records. Eligible patients were divided into the non‐AL and AL groups, and the clinical outcomes were compared between the two groups. The clinical stage of esophageal cancer was classified according to the 8th TNM classification of the Union for International Cancer Control.^15^

### 2.2 Surgical procedures

First, transthoracic esophagectomy and mediastinal lymph‐node dissection using open or thoracoscopic procedures were performed. A hand‐assisted maneuver in the supine position was used to perform laparoscopic gastric mobilization and abdominal lymphadenectomy. The right gastroepiploic artery (RGEA) and the right gastric artery were preserved routinely, and cervical lymph nodes were dissected for thoracic esophageal SCC. A 3.5‐cm‐wide gastric tube along the greater curvature, which was hemodynamically evaluated using TI, was pulled through the neck via the retrosternal route. The triangulating stapling technique (posterior wall inversion and anterior wall eversion) was chosen as the standard procedure for cervical esophagogastric anastomosis. Feeding jejunostomy was routinely performed.

### 2.3 Calculation of anatomical index (AI)

**FIGURE 1:**
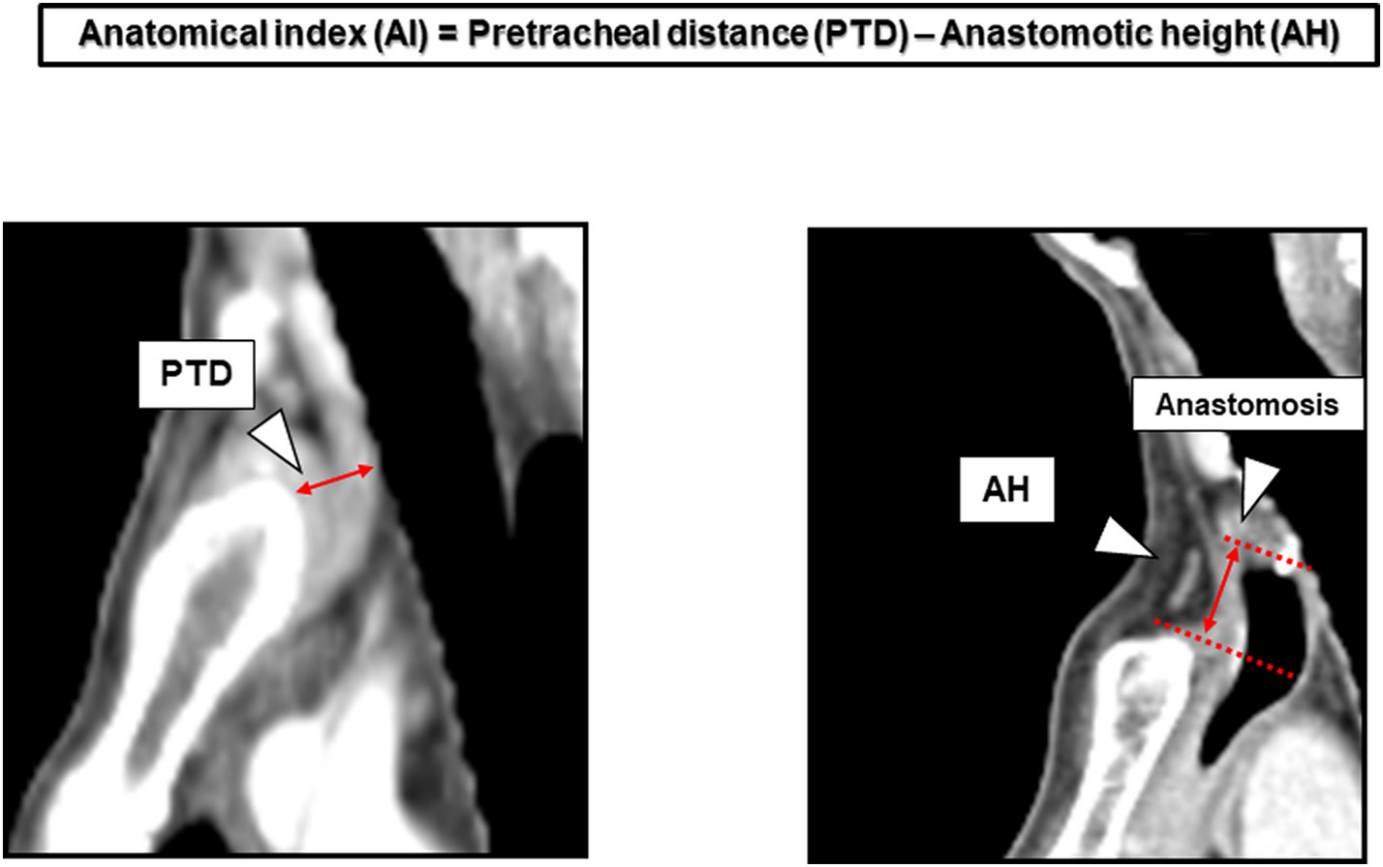
Measurement of the anatomical index (AI). PTD: Narrow space of the thoracic inlet may compress the gastric conduit. AH: The proximal region of the gastric conduit may cause poor vascularization owing to external compression at the thoracic inlet

AI was derived from two anatomical factors: the pretracheal distance (PTD) and anastomotic height (AH) (Figure 1). PTD was measured as the shortest distance from the suprasternal notch to the anterior tracheal wall on sagittal CT images before surgery (Figure S2). AH was measured as the distance from the highest point of the anastomosis to the suprasternal notch on sagittal CT images after surgery (Figure S3). AH was defined as (+) or (−) cm when the anastomosis was above or below the suprasternal notch, respectively. Finally, AI was calculated as follows: AI = PTD − AH.

### 2.4 Hemodynamic assessment of the gastric conduit

**FIGURE 2:**
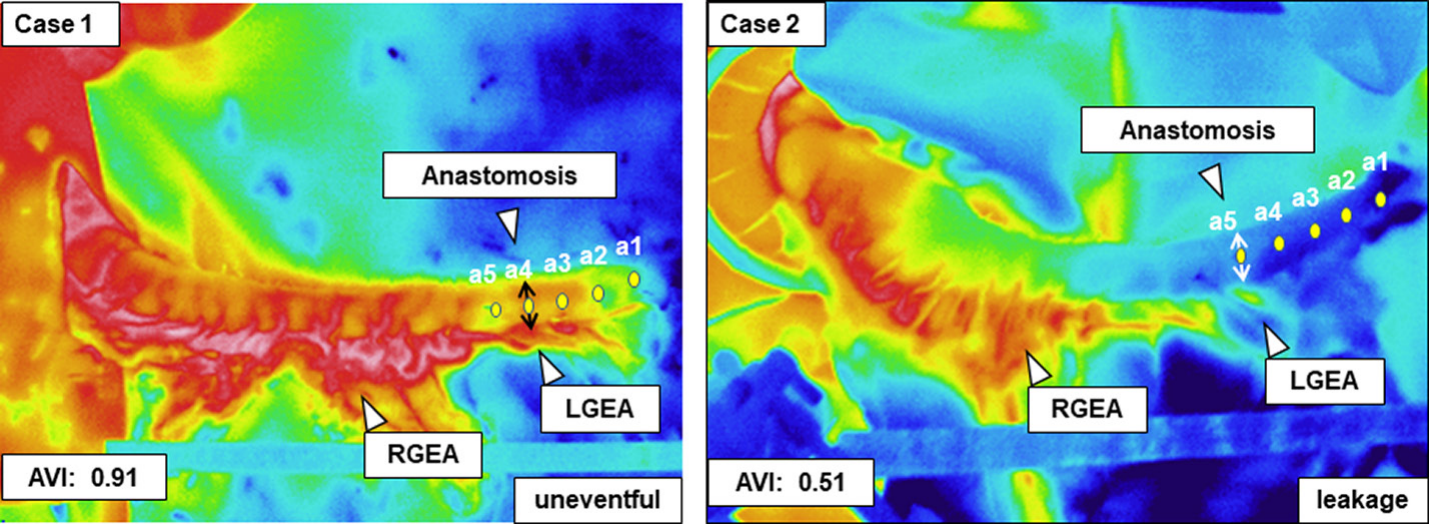
Two representative thermography images. High entire gastric conduit temperature resulted in excellent anastomotic viability index (AVI: 0.91) with uneventful anastomotic complications (Case 1). Conversely, a prominent temperature drop in the proximal region of the conduit resulted in a low AVI score (0.51), and consequently developed anastomotic leakage (Case 2)

First, the entire length of the gastric conduit from the proximal end to the pylorus and total RGEA length were measured. Second, five marking sutures (a1–a5) were placed on the surface of the conduit for the expected anastomosis at 2‐cm intervals from the tip of the conduit. A thermal camera (TVS‐700; Nippon Avionics, Kanagawa, Japan) was used to capture thermal images of the gastric tube at a constant room temperature of 25°C. The mean temperature of the area supplied by RGEA was measured as the reference temperature. Similarly, the average temperatures between the lesser and greater curvatures of the conduit at the five suture marks (a1–a5) were considered as the expected anastomotic temperatures. Fourth, all acquired data were calculated as an objective assessment of graft hemodynamics, and the viability of each predicted anastomotic site was quantified to determine the AVI (Figure 2). Briefly, AVI was calculated using two factors: the RGEA (RGEAr) length ratio, which is the length of the RGEA divided by the distance from the pyloric ring to the predicted anastomosis, and the temperature retention rate (TRr), which is the expected anastomotic temperature divided by the reference conduit temperature. Finally, each AVI was calculated as RGEAr multiplied by TRr (Figures S2 and S4).

### 2.5 Postoperative management and definition of postoperative AL

All patients were carefully monitored for postoperative physical status by performing routine CT on postoperative day (POD) 4. Additional CT was performed in patients with suspected thoracic or abdominal complications. Enteral feeding commenced from jejunostomy on POD 1. Oral intake was originally resumed on POD 7 in the first period, whereas the resumption of oral feeding was changed to POD 4 according to the enhanced recovery protocol in the later period. AL was defined on the basis of clinical signs, such as discharge of pus or saliva from the cervical wound or drain, and findings of extra lumination by postoperative esophagography.

### 2.6 Statistical analysis

Data are presented as medians (interquartile ranges) or events (%). The unpaired Student's *t*‐test or Mann–Whitney *U*‐test were performed to evaluate continuous variables, depending on data normality. The chi‐squared test or Fisher's exact test were performed to evaluate categorical variables. The Youden Index obtained from the receiver operating characteristic curve was used to derive the cutoff value for continuous variables. Logistic regression models were used to determine the risk factors for AL. Statistical significance was set at *P <* 0.05.

Stata/IC (STATA Statistical Software, v. 14.0; StataCorp, College Station, TX, USA) was used to perform all statistical analyses.

## 3 RESULTS

### 3.1 Patient background data (Table 1)

**TABLE 1:** Preoperative characteristics

Variables	Anastomotic leakage (−)	Anastomotic leakage (+)	*P‐v*alue
	*n* = 179	*n* = 23	
Age ≥68 (y)	80 (44.7)	14 (60.9)	0.18
Gender			
Male	155 (86.6)	16 (69.6)	0.06
Female	24 (13.4)	7 (30.4)	
Preoperative BMI ≥23.6 (kg/m^2^)	43 (24.0)	12 (52.2)	0.01
Preoperative albumin <4.0 (g/dL)	97 (54.2)	14 (60.9)	0.80
ASA‐PS 3	6 (3.4)	2 (8.7)	0.23
Comorbidity (diabetes)	14 (7.8)	4 (17.4)	0.13
Preoperative treatment			
None/ESD	83 (46.4)	7 (30.4)	0.18
Chemotherapy or chemoradiation	96 (53.6)	16 (69.6)	
Histologic subtype			
Squamous cell carcinoma	165 (92.2)	22 (95.7)	1
Others	14 (7.8)	1 (4.3)	
Tumor location			
Upper third	19 (10.6)	5 (21.7)	0.16
Middle or lower third	160 (89.4)	18 (78.3)	
Clinical tumor stage (UICC 8th)			
Depth of tumor			
cT1‐2	84 (46.9)	13 (56.5)	0.51
cT3‐4	95 (53.1)	10 (43.5)	
Lymph node metastasis			
cN0	84 (46.9)	9 (39.1)	0.51
cN1‐3	95 (53.1)	14 (60.9)	
Clinical stage			
cStageI‐II	101 (56.4)	14 (60.9)	0.82
cStageIII‐IV	78 (43.6)	9 (39.1)	

Abbreviations: ASA‐PS, American Society Anesthesiologists‐Physical Status; BMI, body mass index; ESD, endoscopic submucosal dissection.

AL developed in 11.4% (23/202) of the study cohort. The patients were divided into the AL (−) (*n* = 179) and AL (+) (*n* = 23) groups. The proportion of patients with a preoperative body mass index (BMI) ≥ 23.6 kg/m^2^ was significantly higher in the AL (+) group than in the AL (−) group (*P =* 0.01). However, other background characteristics and tumor stages were comparable between the two groups.

### 3.2 Assessment of anatomical factors for AL (Figure S5)

Receiver operating characteristic curves were used to evaluate three anatomical factors—PTD, AH, and AI—as potential risk factors for AL. The area under the curve for AI was the highest at 0.790 and 0.731 and 0.586 for AH and PTD, respectively.

### 3.3 Comparison of results between AL (+) and AL (−)

#### 3.3.1 Anatomical and vascular factors (Table 2).

**TABLE 2:** Anatomical and vascular factors in retrosternal gastric reconstruction

Variables	Anastomotic leakage (−)	Anastomotic leakage (+)	*P‐v*alue
	*n* = 179	*n* = 23	
Anatomical factors			
PTD <16.6 (mm)	108 (60.3)	18 (78.3)	0.11
AH ≥13.1 (mm)	63 (35.2)	16 (69.6)	<0.01
AI <1.4	55 (30.7)	19 (82.6)	<0.01
Vascular factors			
RGEAr <0.73	46 (25.7)	13 (56.5)	<0.01
TRr <0.84	37 (20.7)	9 (39.1)	0.06
AVI <0.62	38 (21.2)	13 (56.5)	<0.01

Abbreviations: AH, anastomotic height; AI, anatomical index; AVI, anastomotic viability index; PTD, pretracheal distance; RGEAr, right gastroepiploic artery length ratio; TRr, anastomotic temperature retain rate.

Overall, a significantly greater number of AH ≥13.1 mm, lower AI <1.4, and AVI <0.62 were observed in the AL (+) group than in the AL (−) group (*P <* 0.01). Conversely, the proportions of patients with a PTD <16.6 mm were comparable between the two groups (*P =* 0.11). Regarding vascular factors, the AL (+) group showed significantly lower RGEAr and AVI values than the AL (−) group (*P <* 0.01).

#### 3.3.2 Intraoperative and postoperative characteristics (Table 3)

**TABLE 3:** Intraoperative and postoperative characteristics

Variables	Anastomotic leakage (−)	Anastomotic leakage (+)	*P‐v*alue
	*n* = 179	*n* = 23	
Type of thoracic surgery			
Thoracoscopic	128 (82.6)	19 (82.6)	0.70
Thoracotomy	46 (25.7)	4 (17.4)	
Transhiatal	5 (2.8)	0	
Anastomotic method			
Triangulating stapling technique	174 (97.2)	20 (87.0)	0.1
Hand‐sewn anastomosis	4 (2.2)	3 (13.0)	
Circular stapling technique	1 (0.6)	0	
Vessel reconstruction	10 (5.6)	2(8.7)	0.63
Operative time ≥617 (min)	28 (15.6)	7 (30.4)	0.09
Blood loss ≥1070 (mL)	12 (6.7)	4 (17.4)	0.09
Postoperative complications			
Pneumonia	28 (15.6)	6 (26.1)	0.24
Recurrent laryngeal nerve palsy	49 (27.4)	8 (34.8)	0.47
Length of hospital stay (days)^a^	20 (17–31)	46 (33–62)	<0.01
In‐hospital mortality	0	0	NE

^a^ Data are presented as median (interquartile range).

The median hospital stay length was significantly longer in the AL (+) group than in the AL (−) group (46 vs 20 d, *P <* 0.01). Other intra‐ and postoperative outcomes were comparable between the two groups.

#### 3.3.3 Risk factors for AL development (Table 4)

**TABLE 4:** Logistic regression model for anastomotic leakage

Variables	Univariate analysis	*P‐v*alue	Multivariate analysis	*P‐v*alue
	Odds ratio (95% CI)		Odds ratio (95% CI)	
Age ≥68 (y)	1.92 (0.79–4.68)	0.15		
Gender (Female)	2.83 (1.05–7.58)	0.04		
Preoperative BMI ≥23.6	3.45 (1.42–8.38)	<0.01	7.97 (2.44–26.00)	<0.01
Preoperative Albumin <4.0 (g/dL)	1.32 (0.54–3.19)	0.55		
ASA‐PS, 3	2.75 (0.52–14.50)	0.23		
Comorbidity (diabetes mellitus)	2.48 (0.74–8.31)	0.14		
Preoperative chemotherapy or radiation	1.98 (0.78–5.04)	0.15		
Cancer type, squamous cell carcinoma	1.87 (0.23–14.90)	0.56		
Tumor location, upper third	2.34 (0.78–7.02)	0.13		
Hand‐sewn anastomosis	6.56 (1.37–31.40)	0.02		
Vessel reconstruction	1.61 (0.33–7.85)	0.56		
Tumor stage				
cT3, 4	0.68 (0.09–1.63)	0.38		
cN positive	1.38 (0.57–3.34)	0.48		
cStageIII, IV	0.83 (0.34–2.02)	0.69		
Operative time ≥617 (min)	2.36 (0.89–6.23)	0.09		
Blood loss ≥1070 (mL)	2.93 (0.86–9.99)	0.09		
PTD <16.6 (mm)	2.37 (0.84–6.66)	0.10		
AH ≥13.1 (mm)	4.21 (1.64–10.80)	<0.01	0.71 (0.20–2.63)	0.61
AI <1.4	10.70 (3.48–33.00)	<0.01	23.90 (5.02–114.00)	<0.01
AVI <0.62	4.62 (1.96–11.90)	<0.01	8.02 (2.57–25.00)	<0.01

Abbreviations: AH, anastomotic height; AI, anatomical index; ASA‐PS, American Society Anesthesiologists‐Physical Status; AVI, anastomotic viability index; BMI, body mass index; PTD, pretracheal distance.

Logistic regression analysis was performed to determine risk factors for postoperative AL. Univariate analysis demonstrated that a preoperative BMI ≥23.6 kg/m^2^, hand‐sewn anastomosis, AH ≥13.1 (mm), AI <1.4, and AVI <0.62 were significant risk factors. In the multivariate analysis, preoperative BMI ≥23.6 kg/m^2^ (odds ratio [OR], 7.97; 95% confidence interval [CI], 2.44–26.00; *P <* 0.01), AI <1.4 (OR, 23.90; 95% CI, 5.02–114.00; *P <* 0.01) and AVI <0.62 (OR, 8.02; 95% CI, 2.57–25.00; *P <* 0.01) were found to be independent risk factors for AL.

#### 3.3.4 Risk stratification of AL (Table 5)

**TABLE 5:** Risk stratification of anastomotic leakage (AL) using AI and AVI

Variables	Incidence of AL	*P‐v*alue	Odds ratio (95% CI)	*P‐v*alue
AI ≥1.4, AVI ≥0.62	2/99 (2.0)	<0.01	References	–
AI ≥1.4, AVI <0.62	2/29 (6.9)		3.59 (0.48–26.70)	0.21
AI <1.4, AVI ≥0.62	8/53 (15.1)		8.62 (1.76–42.20)	<0.01
AI <1.4, AVI <0.62	11/21 (52.4)		53.30 (10.30–275.00)	<0.01

Abbreviations: AI, anatomical index; AVI, anastomotic viability index.

The patients were stratified into the following four AL risk groups according to the AI and AVI values: the low‐risk group included patients with AI ≥1.4 and AVI ≥0.62 (2/99, 2.0%); the intermediate low‐risk group included patients with AI ≥1.4 and AVI <0.62 (2/29, 6.9%); the intermediate high‐risk group included patients with AI <1.4 and AVI ≥0.62 (8/53, 15.1%); and the high‐risk group included patients with AI <1.4 and AVI <0.62 (11/21, 52.4%). The incidence of AL was significantly different between the groups (*P <* 0.01). The AL risk did not differ significantly between the low‐risk and intermediate low‐risk patients (OR, 3.59; 95% CI, 0.48–26.70; *P =* 0.21). In contrast, the AL risk was significantly higher in the intermediate high‐risk patients (*P <* 0.01; OR, 8.62; 95% CI, 1.76–42.20) and high‐risk patients (*P <* 0.01; OR, 53.30; 95% CI, 10.30–275.00) than in the low‐risk patients.

## 4 DISCUSSION

Although several previous studies have examined various factors associated with AL, there is no doubt that inadequate gastric conduit perfusion is fundamentally associated with the primary cause of the underlying anastomotic impairment. Poor vascularization of the gastric conduit is affected not only by insufficient blood flow in the conduit but also by anatomical compression of the gastric conduit in the reconstruction route.^13^, ^14^ In the present study, we found that AI and AVI were independent risk factors for AL. Furthermore, we stratified the patients into four groups according to their AL risk. This approach can enable surgeons to select either the retrosternal or PM route during surgery and minimize the incidence of AL.

Regarding anatomical restriction, Anegg et al revealed that the interstitial partial pressure of oxygen at an esophagogastric anastomosis was higher in the posterior mediastinal route than in the retrosternal rout.^16^ Sato et al showed the clinical significance of AL with a low PTD/sternum–vertebral body distance in the retrosternal route.^13^ Other previous studies also have found that compression of the gastric conduit was associated with AL regardless of the reconstruction route.^14^ Kunisaki et al reported that the thoracic inlet should exceed 700 mm^2^ to prevent anastomotic complications when the retrosternal route is used. They suggested partial resection of the left‐clavicular head and manubrium of the sternum in the case of a narrow thoracic inlet.^12^

Similar to previous studies, we found that AI, as an anatomical factor, was significantly associated with the incidence of AL. In this context, we previously reported the clinical relationship between AL, PTD, and AH. Nishikawa et al stated that higher esophagogastric anastomosis led to a long gastric conduit at the cervical space, which may cause low blood flow at the anastomosis due to anatomical restriction at the thoracic inlet. In contrast, PTD did not show a significant result in the study; however, since previous studies have shown that a tight thoracic inlet in the retrosternal tract affects gastric vascular impairment, PTD may have similar relevance. Given these findings, although hemodynamic insufficiency caused by anatomical restriction is not clearly proven, calculation using PTD and AH for AI is considered logical and more remarkably associated with the development of AL. Currently, AH is measured from postoperative CT images, whereas the reconstruction route and necessity of expansion of the pretracheal space can be chosen by the appropriate AH, which can be assumed from a tumor location before surgery.

Blood flow in the gastric conduit has been investigated using various modalities, such as indocyanine green angiography or TI. Previously, we reported that the use of RGEAr and TRr with TI to calculate AVI could quantitatively predict gastric perfusion. Although the RGEA length is the known dominant factor for gastric conduit perfusion, the intramural vascular network has another critical role in gastric perfusion, which should also be taken into account for accurate hemodynamic assessment.^9^

Venous congestion can cause vascular impairment of the gastric conduit, similar to arterial ischemia. Tsutsumi et al demonstrated that gastric conduit ischemia with congestion was significantly associated with AL development, but not with conduit ischemia alone.^17^ We found that TI represents the same low temperature in both tissue ischemia and congestion, which can be translated as poor tissue perfusion.^9^ Furthermore, liver cirrhosis or fibrosis, known to cause gastric‐venous congestion, was reported to correlate with AL development after esophagectomy, suggesting that gastric conduit congestion is associated with low perfusion.^18^, ^19^

Several procedures, such as the Kocher maneuver, adhesiolysis between the stomach and pancreas, and an extended gastric conduit, have been recommended to shift the gastric conduit cranially for secure perfusion at the anastomotic site. However, the gastric conduit is usually sufficiently long for esophagogastric anastomosis, which is thus conducted when the gastric conduit is too short for an appropriate anastomotic site derived by TI. Moreover, microvascular augmentation can be the final option (supercharge and/or superdrainage) for patients at high risk of AL.^20^

In addition to anatomical and vascular factors, preoperative BMI is also associated with an increased incidence of AL. A meta‐analysis by Mengardo et al reported that a high BMI was a risk factor for AL in patients who had been esophagectomized,^21^ and the rate of diabetes was higher in patients with a high BMI than in those with a normal BMI. Okamura et al demonstrated that a high glycated hemoglobin level was correlated with the incidence of AL.^22^ These associations might be explained by impaired glucose tolerance, which can lead to delayed wound healing due to microvascular, immunological, and biochemical abnormalities.^23^, ^24^, ^25^

According to the multivariate analyses (Table 4), we used AI and AVI to stratify the patients at risk of AL into low‐, intermediate‐low‐, intermediate‐high‐, and high‐risk groups for AL. The AL in the high‐risk group was extremely high (52.4%). Hence, PM reconstruction or partial clavicle and manubrium resection is recommended to prevent external compression of the gastric conduit during retrosternal reconstruction. Moreover, the subcutaneous route may be an option in cases with a poor overall condition or in those where there is a risk of fatality due to AL or gastric necrosis within the mediastinum. Patients in the intermediate, high‐, and low‐risk groups would also require changes in countermeasures. In the patients with intermediate high‐risk, the AL risk was higher for the AI factor than for the AVI factor; therefore, the use of alternative reconstruction routes or thoracic inlet expansion is recommended. Alternatively, poor vascularization of the conduit is considered a leading cause of AL in intermediate low‐risk patients, suggesting a change in the anastomosis to a more proximal site of the conduit for these patients. Additional microvascular anastomosis would be another option if that is not applicable.

We also conducted an additional analysis to assess the correlation between the number of risk factors (AVI <0.62, AI <1.4, and BMI ≥23.6) and the development of AL (Figure S6). Surprisingly, patients without these risk factors did not develop AL. The incidence of AL increased as the number of risks increased (range: 0%–75%). Given these facts, prevention of both local (e.g. AI and AVI) and systemic (e.g. obesity) factors is essential to eradicate the development of AL.

This study had several limitations. First, this was a retrospective study conducted at a single institution in a small cohort. Second, postoperative CT images were used to assess the AI. Therefore, future studies should use preoperative CT images to determine the AI.

## 5 CONCLUSION

The study results showed that AI and AVI were powerful predictors of AL. Additionally, the use of AI and AVI enabled the stratification of the risk of AL in patients who underwent esophagectomy with retrosternal reconstruction.

## AUTHOR CONTRIBUTIONS

KT designed this research, acquired the data, and drafted the article. YT revised the article. All Authors read and approved the final article.

## FUNDING INFORMATION

None.

## CONFLICT OF INTEREST STATEMENT

The Authors declare that they have no competing interests in relation to this study.

## ETHICS STATEMENT

Approval of the research protocol: This study protocol was approved by the Institutional Review Board of the Jikei University School of Medicine (33–303).

Informed consent: The need for informed consent was waived because of the retrospective design of the study.

Registry and the Registration No. of the study: N/A.

Animal Studies: N/A.

## Supporting information


Figures S1–S6

## Data Availability

The data that support the findings of this study are available from the corresponding author, KT, upon reasonable request.
