# Supplementary material for: Anatomical and anastomotic viability indexes for stratifying the risk of anastomotic leakage in esophagectomy with retrosternal reconstruction
Source: Ann Gastroenterol Surg. 2023 May 16;7(6):896–903. doi: 10.1002/ags3.12693 (PMC10623953; doi:10.1002/ags3.12693)
Supplement: Supplementary file 1 — Figures S1–S6 [file AGS3-7-896-s001.pptx]

## Slide 1
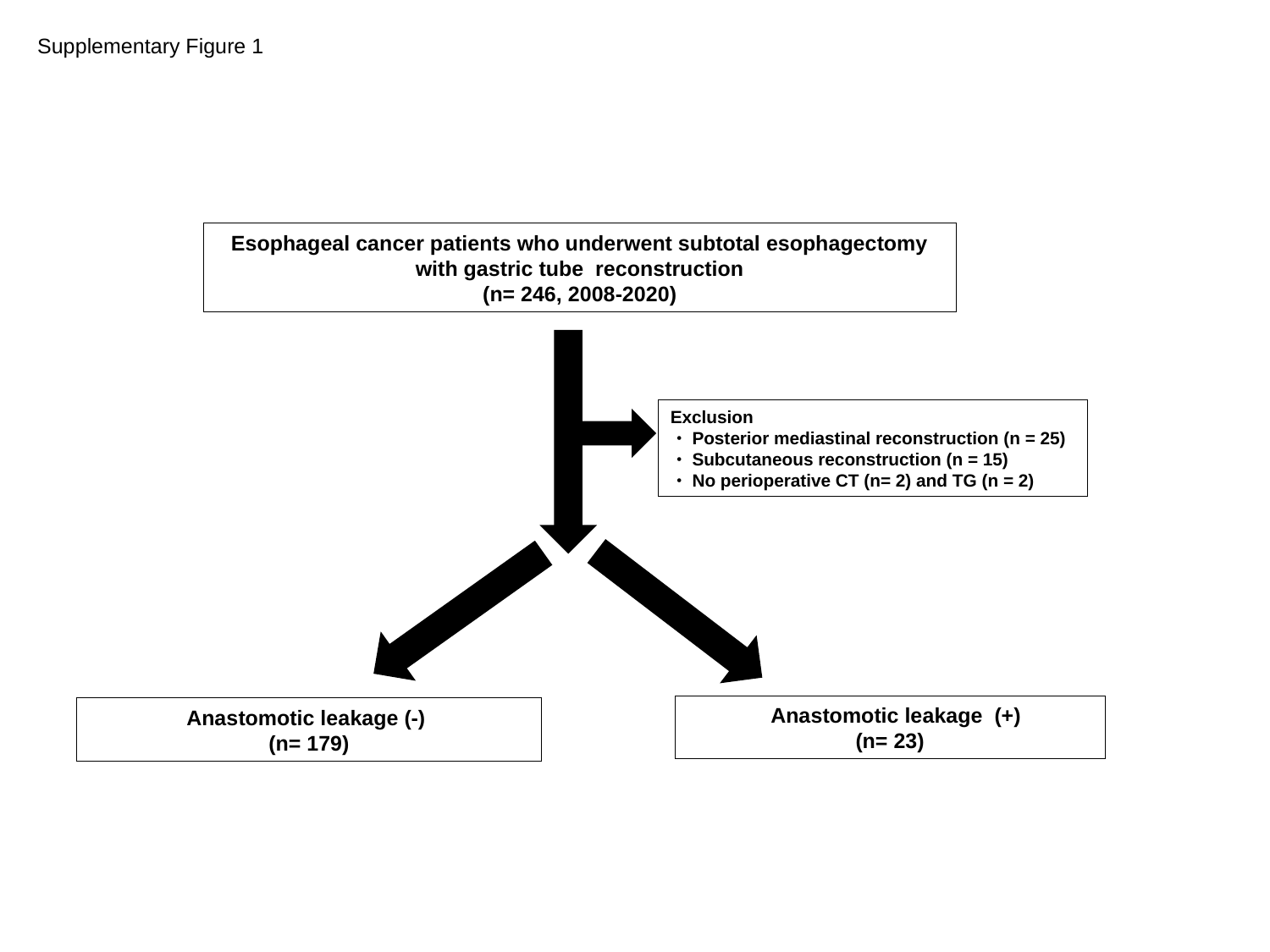

Supplementary Figure 1
Esophageal cancer patients who underwent subtotal esophagectomy with gastric tube reconstruction
(n= 246, 2008-2020)
Exclusion
・Posterior mediastinal reconstruction (n = 25)
・Subcutaneous reconstruction (n = 15)
・No perioperative CT (n= 2) and TG (n = 2)
 Anastomotic leakage (+)
(n= 23)
Anastomotic leakage (-)
(n= 179)

## Slide 2
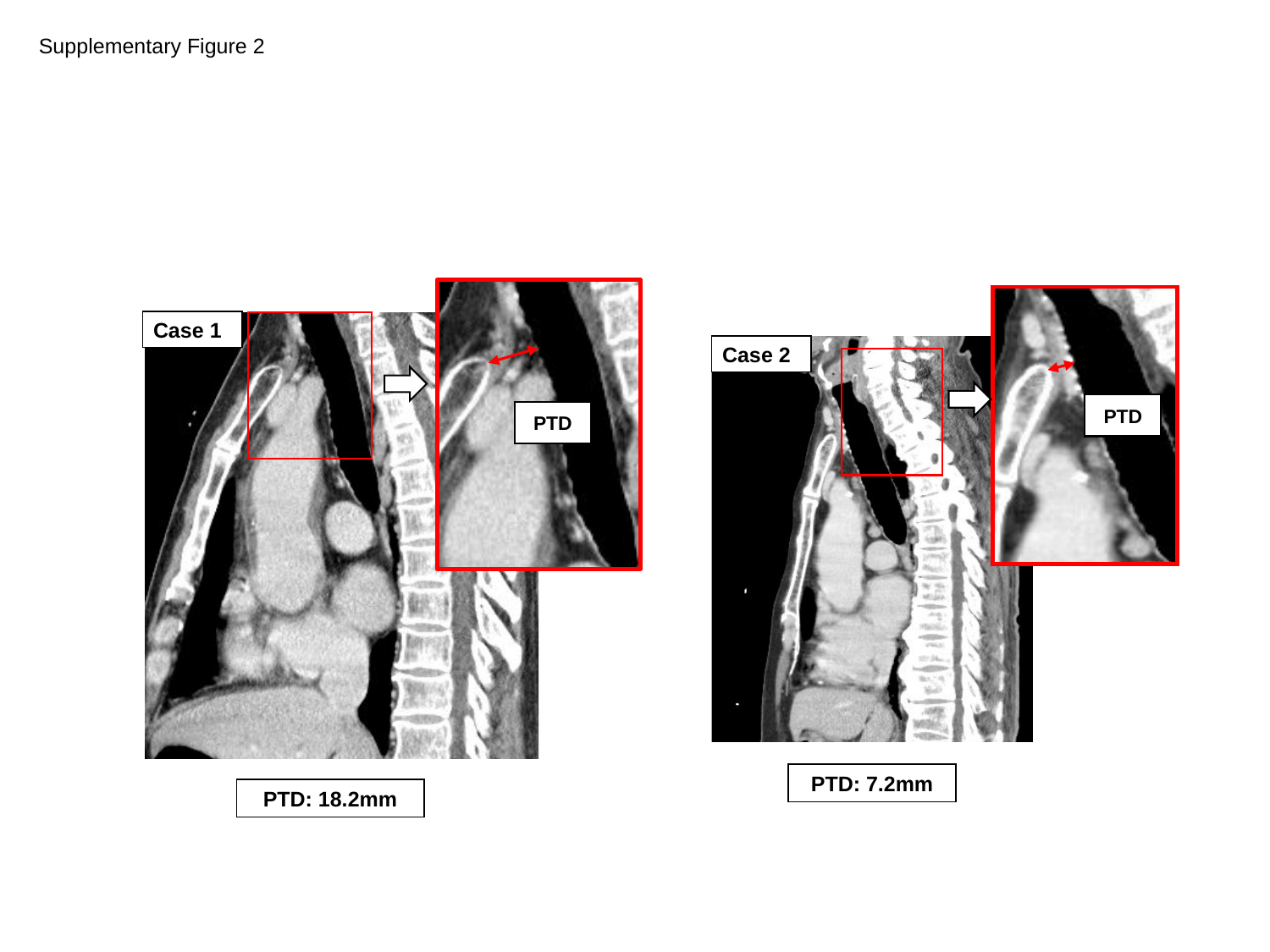

Supplementary Figure 2
Case 1
Case 2
PTD
PTD
STD
PTD: 7.2mm
PTD: 18.2mm

## Slide 3
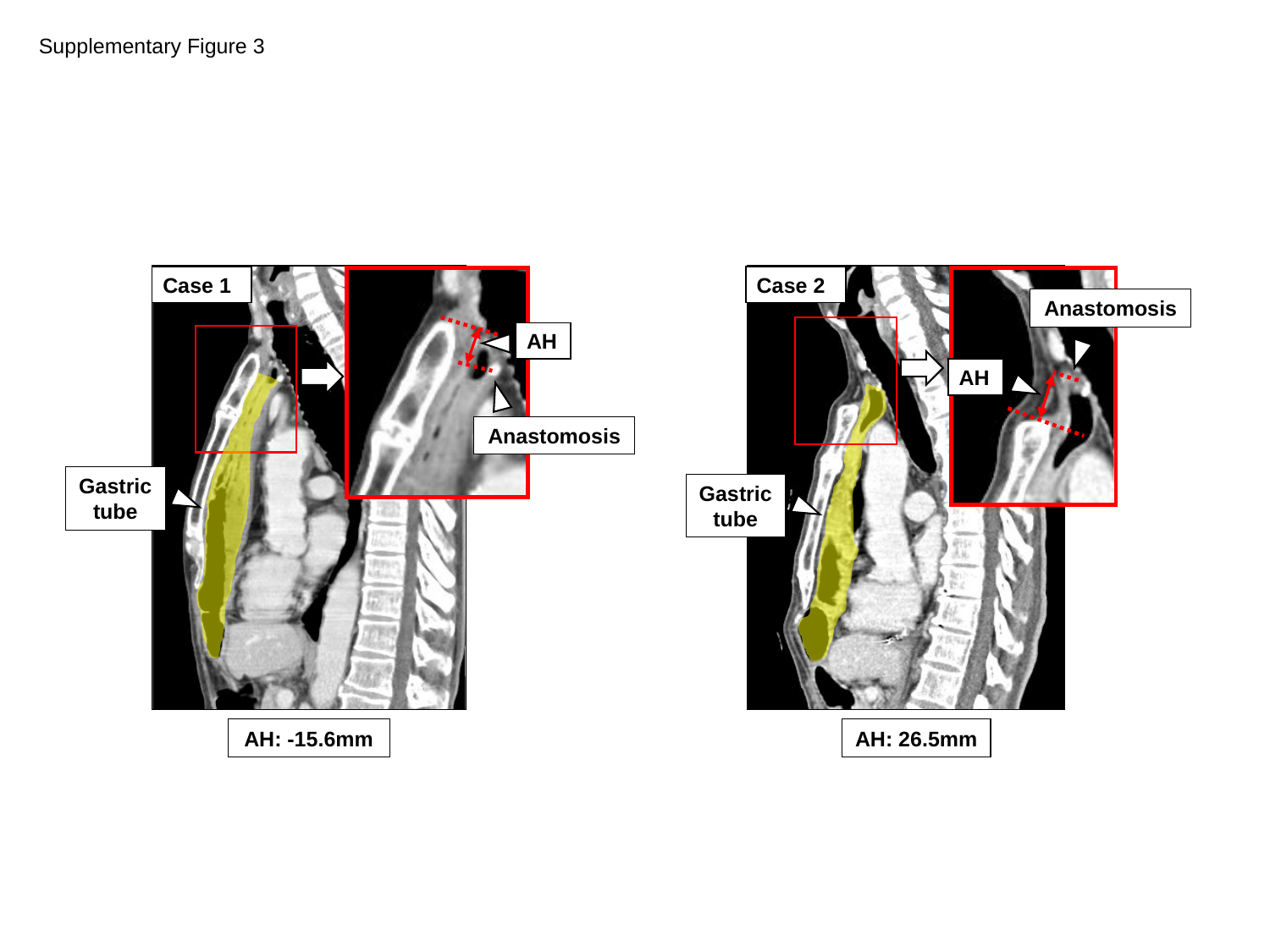

Supplementary Figure 3
Case 1
Case 2
Anastomosis
AH
AH
Anastomosis
Gastric
tube
Gastric
tube
AH: -15.6mm
AH: 26.5mm

## Slide 4
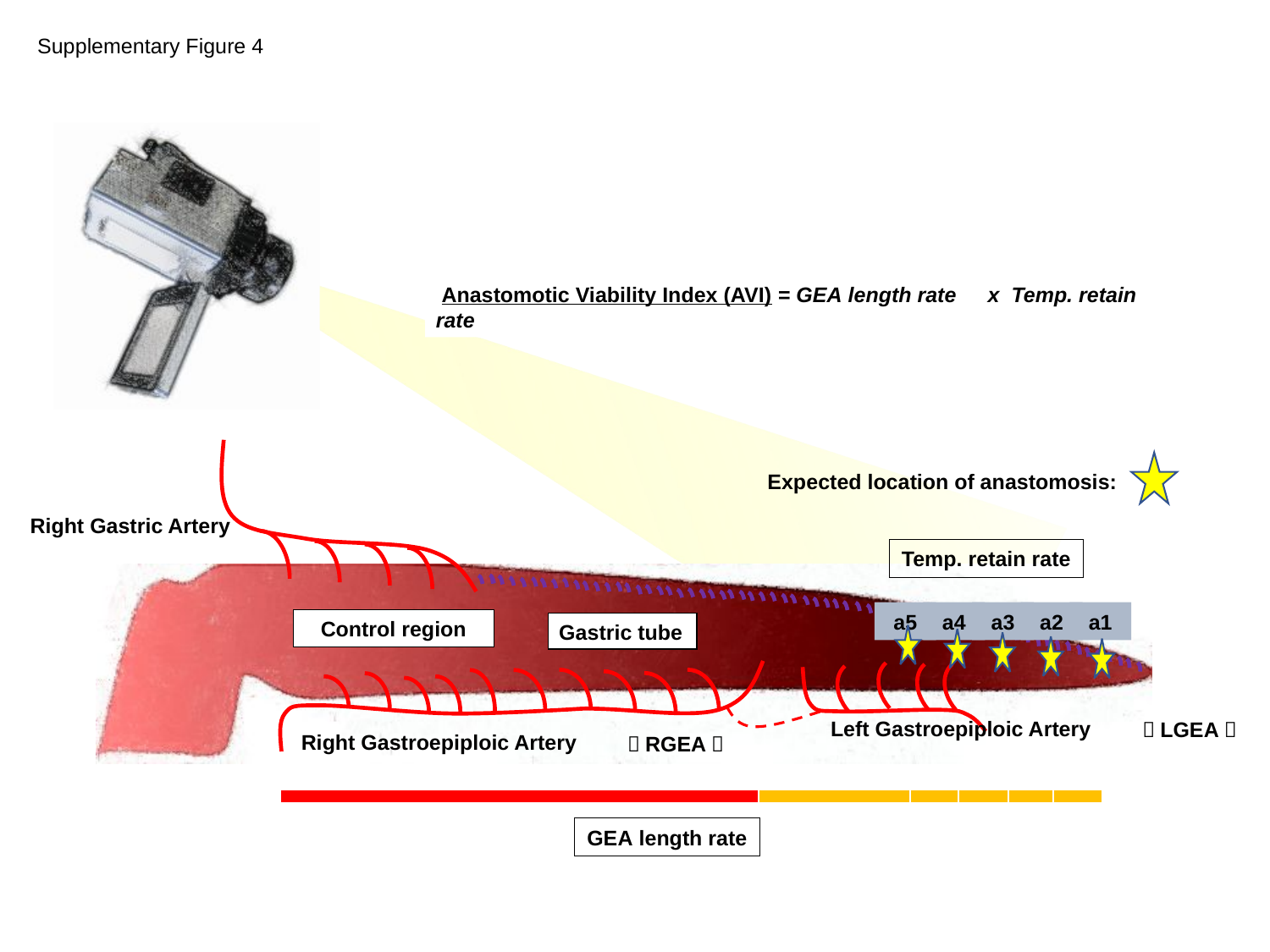

Supplementary Figure 4
 Anastomotic Viability Index (AVI) = GEA length rate　x Temp. retain rate
Expected location of anastomosis:
Right Gastric Artery
Temp. retain rate
a5
a4
a3
a2
a1
Control region
Gastric tube
Left Gastroepiploic Artery
（LGEA）
Right Gastroepiploic Artery
（RGEA）
GEA length rate

## Slide 5
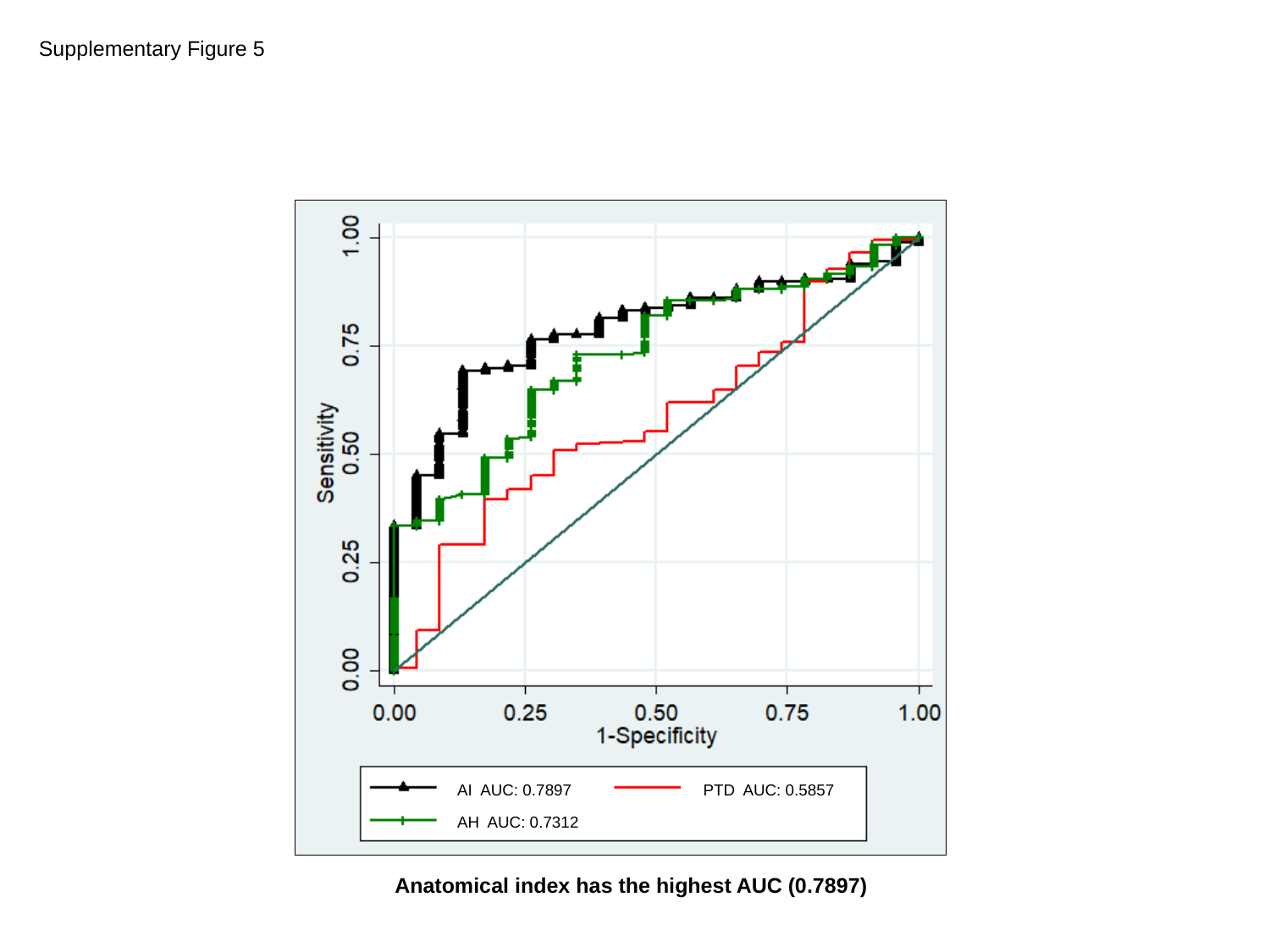

Supplementary Figure 5
AI AUC: 0.7897
PTD AUC: 0.5857
AH AUC: 0.7312
Anatomical index has the highest AUC (0.7897)

## Slide 6
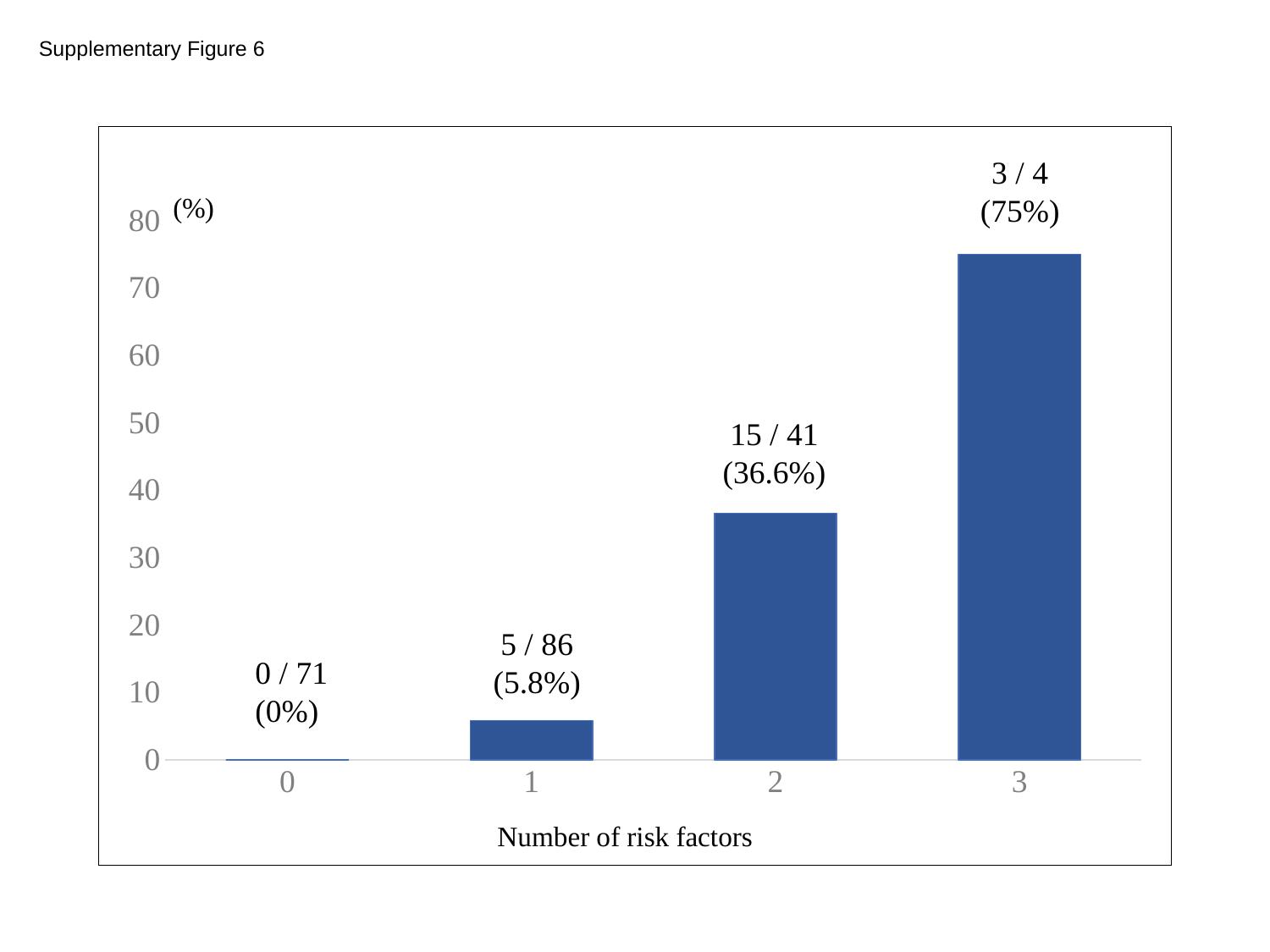

Supplementary Figure 6
### Chart
| Category | AL率 |
|---|---|
| 0 | 0.0 |
| 1 | 5.8 |
| 2 | 36.6 |
| 3 | 75.0 |3 / 4
(75%)
15 / 41
(36.6%)
5 / 86
(5.8%)
0 / 71 (0%)
Number of risk factors
